# Nerve Growth Factor and Diabetic Neuropathy

**DOI:** 10.1155/EDR.2003.271

**Published:** 2003

**Authors:** Gary Pittenger, Aaron Vinik

**Affiliations:** 1 The Leonard Strelitz Diabetes Institutes Department of Internal Medicine and Department of Pathology/Neurobiology Eastern Virginia Medical School Norfolk Virginia USA

## Abstract

Neuropathy is one of the most debilitating complications of both type 1 and type 2 diabetes, with estimates of prevalence between 50–90% depending on the means of detection. Diabetic neuropathies are heterogeneous and there is variable involvement of large myelinated fibers and small, thinly myelinated fibers. Many of the neuronal abnormalities in diabetes can be duplicated by experimental depletion of specific neurotrophic factors, their receptors or their binding proteins. In experimental models of diabetes there is a reduction in the availability of these growth factors, which may be a consequence of metabolic abnormalities, or may be independent of glycemic control. These neurotrophic factors are required for the maintenance of the neurons, the ability to resist apoptosis and regenerative capacity. The best studied of the neurotrophic factors is nerve growth factor (NGF) and the related members of the neurotrophin family of peptides. There is increasing evidence that there is a deficiency of NGF in diabetes, as well as the dependent neuropeptides substance P (SP) and calcitonin gene-related peptide (CGRP) that may also contribute to the clinical symptoms resulting from small fiber dysfunction. Similarly, NT3 appears to be important for large fiber and IGFs for autonomic neuropathy. Whether the observed growth factor deficiencies are due to decreased synthesis, or functional, e.g. an inability to bind to their receptor, and/or abnormalities in nerve transport and processing, remains to be established. Although early studies in humans on the role of neurotrophic factors as a therapy for diabetic neuropathy have been unsuccessful, newer agents and the possibilities uncovered by further studies should fuel clinical trials for several generations. It seems reasonable to anticipate that neurotrophic factor therapy, specifically targeted at different nerve fiber populations, might enter the therapeutic armamentarium.


*Experimental Diab. Res.*, 4:271–285, 2003
Copyright © Taylor and Francis Inc.
ISSN: 1543-8600 print / 1543-8619 online
DOI: 10.1080/15438600390249718

### Nerve Growth Factor and Diabetic Neuropathy

**Gary Pittenger and Aaron Vinik**

*The Leonard Strelitz Diabetes Institutes, Department of Internal Medicine, and Department of Pathology/Neurobiology, Eastern Virginia Medical School, Norfolk, Virginia, USA*

**Neuropathy is one of the most debilitating complications of both type 1 and type 2 diabetes, with estimates of prevalence between 50–90% depending on the means of detection. Diabetic neuropathies are heterogeneous and there is variable involvement of large myelinated fibers and small, thinly myelinated fibers. Many of the neuronal abnormalities in diabetes can be duplicated by experimental depletion of specific neurotrophic factors, their receptors or their binding proteins. In experimental models of diabetes there is a reduction in the availability of these growth factors, which may be a consequence of metabolic abnormalities, or may be independent of glycemic control. These neurotrophic factors are required for the maintenance of the neurons, the ability to resist apoptosis and regenerative capacity. The best studied of the neurotrophic factors is nerve growth factor (NGF) and the related members of the neurotrophin family of peptides. There is increasing evidence that there is a deficiency of NGF in diabetes, as well as the dependent neuropeptides substance P (SP) and calcitonin gene-related peptide (CGRP) that may also contribute to the clinical symptoms resulting from small fiber dysfunction. Similarly, NT3 appears to be important for large fiber and IGFs for autonomic neuropathy. Whether the observed growth factor deficiencies are due to decreased synthesis, or functional, e.g. an inability to bind to their receptor, and/or abnormalities in nerve transport and processing, remains to be established. Although early studies in humans on the role of neurotrophic factors as a therapy for diabetic neuropathy have been unsuccessful, newer agents and the possibilities uncovered by further studies should fuel clinical trials for several generations. It seems reasonable to anticipate that neurotrophic factor therapy, specifically targeted at different nerve fiber populations, might enter the therapeutic armamentarium.**

**Keywords** Autonomic; C-Fibers; Large Fibers; Neurotrophins; NGF; Regeneration

### INTRODUCTION

One of the most insidious complications of diabetes is peripheral neuropathy. Diabetic neuropathy commonly presents as a small fiber sensory neuropathy exhibiting the typical "stocking and glove" distribution. It is the major cause of amputations in the United States, accounting for 50% to 70% of all nontraumatic amputations, and diabetic neuropathy accounts for more hospitalizations than all other diabetic complications combined (Caputo et al., 1994; Greene et al., 1992; Vinik et al., 1995a). Thus, discovery of an effective treatment would be a significant boon to the care of diabetes patients.

The heterogeneity of diabetic neuropathy, with involvement of large, myelinated fibers that provide proprioception, touch/pressure perception, and motor innervation, as well as small, thinly or nonmyelinated fibers providing temperature and pain perception along with autonomic function, suggests multiple factors in pathogenesis. The most common pathology in both the nerves serving the periphery and in the skin itself is a loss of nerve fibers. Efforts to determine the pathogenetic mechanisms leading to peripheral nerve dysfunction in diabetes has proven quite controversial. There is good evidence for several mechanisms playing a key role leading to neuronal loss in diabetes, including: (1) metabolic disruption resulting from chronic hyperglycemia, including increased sorbitol pathway involvement and accumulation of advanced glycation end products (AGEs); (2) altered vascular function leading to loss

Received 18 February 2003; accepted 11 April 2003.
This work was supported by grants from the American Diabetes Association (ADA), HUD, NASA, The Diabetes Institutes Foundation, and pharmaceutical industry.
Address correspondence to Gary L. Pittenger, PhD, The Strelitz Diabetes Institutes, 855 West Brambleton Avenue, Norfolk, VA 23510, USA. E-mail: pittengl@evms.edu of nutritive support for peripheral nerve fibers; (3) altered neurotrophic/growth factor availability (some aspects addressed here); (4) autoimmune processes leading to disrupted neuronal function; (5) oxidative stress causing neuronal loss and dysfunction. It is clear from the 10-year Diabetes Control and Complications Trials that hyperglycemia is an important component driving neuronal loss (DCCT Research Group, 1993); however, it seems likely that each of these mechanisms can contribute to the degeneration of peripheral nerve function in any given patient. Determination of the mechanisms active in a particular patient might provide better insight into the appropriate means of treatment of the syndrome. This range of pathologies contributing to diabetic neuropathy in patients might also help explain the difficulties in translating mechanisms and treatments discovered in animal models to the human condition.

Early in the development of diabetic neuropathy, there appears to be an attempt at axonal regeneration, remyelination, and synaptogenesis, possibly mediated by growth factors, that ultimately fails to restore function. This failure is accompanied by an enhanced rate of programmed cell death, or apoptosis. Further supporting a role for disruption of normal growth factor function are studies showing that many of the neuronal abnormalities seen in diabetes can be duplicated by experimental depletion of specific growth factors, their receptors, or their binding proteins. It has been reported that there is a reduction in specific growth factors in animal models of diabetes as well as in humans, some of which are reviewed in this issue. These reductions may be due to the associated metabolic abnormalities or they may be independent of glycemic status. Many of these factors are directly acting to maintain the health of neurons, by conferring the ability to resist apoptosis and promoting regeneration. The best studied of these factors is nerve growth factor (NGF). NGF is part of a family of peptides known to exert neurotrophic effects. It seems clear that there is a reduction of NGF in diabetes, with evidence for deficiencies in the other neurotrophins, as well as in neurotransmitters such as substance P (SP) and calcitonin gene–related peptide (CGRP). These losses can all contribute to clinical perturbations in small fiber function. The clinical syndrome can also be generated by selective deletion of low-affinity NGF receptors in genetically altered transgenic animals. Clinical trials with NGF have proven disappointing, but there remain questions regarding the design of the studies. We believe that NGF still hold promise for treating sensory and autonomic neuropathies.

In summary, there is strong support for the hypothesis that reduced levels or activity of NGF play a significant role in the pathogenesis of diabetic neuropathy. Whether these deficiencies are a result of lower systemic levels, an inability to activate appropriate receptors, or abnormalities in postreceptor activity remains to be determined. Although studies of NGF in animal models suggest a significant role, clinical studies have been unsuccessful. Careful analysis of the problems with these early clinical trials may lead to a better understanding of the utility of NGF in treating diabetic neuropathy. We anticipate that NGF will remain a viable approach in seeking therapies for diabetic neuropathy.

### ANIMAL MODELS USED TO STUDY DIABETIC NEUROPATHY

There are a number of rodent models that have been employed to study the pathogenesis of diabetic neuropathy, including models of type 2 and type 1 diabetes as well as drug-induced diabetes. In early studies of diabetes, it was difficult to establish neuropathic changes in animal models, but with persistence and modern techniques, it has been possible to look at biochemical, electrophysiological, and morphological changes over time and get a better appreciation for the pathogenesis of diabetic neuropathy in all of these models. Examples of the models for type 2 diabetes used in studies of diabetic neuropathy include the db/db mouse, which has a relatively severe disease (Schmidt et al., 1995), the ob/ob mouse, used less frequently, and the "Zucker" fatty (fa/fa) rat. Mouse models are limited in studies of neuropathy due to size constraints of the nerves, thus most of the data in the field has been collected using the larger rats, and there has been a good deal of useful data collected using rat models in studies of diabetic neuropathy. In addition, there are nutritionally induced type 2 models such as the overfed C57Blk/6J mouse, in which physiology can be studied. Because the prevalence of type 2 diabetes in humans is approximately 90%, these models have proven important in the studies of diabetic complications. In human type 2 diabetes, neuropathy often precedes the diagnosis of diabetes, making it difficult to stage these patients. It is hoped that animal studies of type 2 diabetes can lead to more meaningful details of the progression of nerve dysfunction in these patients.

Less well studied are the models of type 1 diabetes such as the NOD mouse (Sango et al., 1994) and the Wistar BioBreeding (BB) rat (Nakhooda et al., 1977, 1978). The line includes both diabetes-prone and diabetes-resistant strains, providing a convenient control group. In addition, the BB/Z rat has proven useful for studies of both types of diabetes due to genetic variants that can also serve as models for type 2 diabetes or as controls (Pierson et al., 2003; Sima et al., 2001).

The most common model used for diabetic neuropathy studies is drug-induced diabetes in rats wherein a drug, usually streptozotocin (STZ), which specifically destroys $\beta$ cells, is given to the animals. STZ can be given neonatally, resulting in a syndrome mimicking type 2 diabetes as the animal ages, or it can be given to the adult in an acute fashion, leading to a type 1

diabetes-like syndrome. The caveat here is that although these animals exhibit hyperglycemia and many of the changes associated with diabetes, they do not endure some of the other physiological changes associated with the development of diabetes, such as autoimmunity or AGE accumulation. Thus, although information can be gained on specific mechanisms related directly to hyperglycemia, the interplay of hyperglycemia with other pathways remains elusive.

Neuropathy in these models is detected by a number of methods. The most common marker is nerve conduction velocity; however, this is mainly an indicator of motor nerve function, whereas diabetic neuropathy most commonly includes sensory deficits. It should also be noted that although animals with diabetes develop a sensory syndrome exhibiting both allodynia and thermal hyperalgesia, this is not the case in humans. The differences may explain problematic results with various forms of treatment that appear successful in animal models, yet disappointing in human trials.

The use of these models in conjunction with the proposed mechanisms of nerve destruction have led to a number of proposed therapies for diabetes patients with neuropathy. To date, most have proven disappointing in clinical trials. However, the most promising remain the growth factors, and of these the prospects for neurotrophins are still bright.

### NGF

NGF was first described in the 1950s by Levi-Montalcini (Levi-Montalcini and Booker, 1960; Levi-Montalcini and Hamburger, 1951) and is the most thoroughly studied of the neurotrophins. Numerous early studies showed the dependence of neural crest-derived cells, sympathetic neurons, and dorsal root ganglion (DRG) sensory neurons on NGF for development. More recent studies have demonstrated that sympathetic and DRG neurons are also dependent on NGF for maintenance (Calcutt et al., 1990) and survival (Rich et al., 1987). These populations are commonly affected in diabetic neuropathy.

Following sciatic nerve section in normal adult rats, a significant number of the axotomized neurons in the involved DRGs die (Calcutt et al., 1990; Himes and Tessler, 1989; Melville et al., 1989). This cell loss can be completely eliminated by the application of exogenous NGF to the cut proximal end of the nerve (Rich et al., 1987). Moreover, those DRG neurons that do not die following sciatic nerve section (Rich et al., 1987) or in vivo NGF depletion (Calcutt et al., 1990) exhibit significant atrophy of their cell bodies and axons. This atrophy is at least partially reversed by the application of exogenous NGF (Rich et al., 1987).

NGF is also an important regulator of SP synthesis in adult DRG neurons (Lindsay and Harmar, 1989; Schwartz et al., 1982). SP is found in the sympathetic nervous system as well as in a subpopulation of DRG neurons. It has been implicated in diverse and widespread activities, including vasodilatation, gut motility, and nociception, all of which are perturbed in diabetic neuropathy. Lindsay and Harmar (1989) have shown that NGF is involved in the regulation of mRNAs that encode the precursor molecules of SP and CGRP. In vitro NGF deprivation caused cultured adult DRG neurons to down-regulate SP and CGRP precursor molecules mRNAs (Lindsay and Harmar, 1989). Moreover, when adult rats were immunized against mouse NGF, a procedure that caused an autoimmune depletion of NGF, SP levels were reduced in the DRG, spinal cord, and skin by approximately 65% (Schwartz et al., 1982). Calcutt and colleagues (1990) concluded that in STZ-diabetic rats, a selective down-regulation of SP precursor gene expression was due to a decline in retrogradely transported NGF reaching the ganglia, which in turn could explain the reduction in SP synthesis and transport found in diabetic rats. It remains to be established, however, whether the reduction in SP is pertinent to the symptom complex of neuropathy.

A number of functional disturbances are found in the dermal microvasculature of diabetic subjects. These include (1) decreased microvascular blood flow; (2) increased vascular resistance, decreased tissue $Po_2$; and (3) altered vascular permeability characteristics, such as loss of the anionic charge barrier and decreased charge selectivity. Decreased microvascular blood flow and increased vascular resistance in diabetes could result from alterations in dermal neurovascular function, such as impaired dilator responses to SP and CGRP and reactivity to nociceptive stimulation. Diabetes also disrupts vasomotion, the rhythmic contraction exhibited by arterioles and small arteries (Benbow et al., 1995; Stansberry et al., 1996). Unmyelinated C-fibers, which constitute the central reflex pathway, are assumed to be damaged in diabetic neuropathy, contributing to abnormalities in cutaneous blood flow (Stansberry et al., 1999). These neurons are dependent on NGF for their integrity and survival (Rich et al., 1987; Vinik et al., 1995b). The effect of NGF depletion may be mediated through down-regulation of neurofilament gene expression or RNAs that encode the precursor molecule of SP or CGRP, both of which are NGF dependent and down-regulated in diabetes (Vinik et al., 1995b).

The major neurotrophin considered here is NGF, although the family, related both structurally and functionally, is much larger, and includes neurotrophin-3, -4, and -5 (NT-3, NT-4, and NT-5), glial-derived neurotrophic factor (GDNF), brain-derived neurotrophic factor (BDNF), and ciliary neurotrophic factor (CNTF). These proteins have diverse actions on distinct populations of developing neurons (Barde et al., 1982; Hallbook et al., 1991; Hohn et al., 1990; Jones and Reichardt, 1990; Maisonpierre et al., 1990; Rosenthal et al., 1990). Other growth factors with direct effects on neurons include insulin, insulin-like growth factors (IGFs), laminin and saposins, cytokines and other growth factor–releasing agents. The concept of neurotrophins, with NGF as the prime example, has led to studies showing their synthesis in target tissues, with delivery to the affected neuron where the signal is delivered through retrograde transport up the axon to exert the trophic and survival effects (Merhi et al., 1998). The interaction of neurons with the growth factors released from target tissues continues throughout life to promote the functioning, maintenance, and survival of both elements. There is neuronal dependence on target cells for neurotrophic factors. In the neuronal soma, these factors regulate gene expression and protein synthesis. In turn, the target tissues are dependent on innervation by the dependent neurons to maintain tissue structure and function.

### NEUROTROPHIN RECEPTORS

Neurotrophins bind two classes of receptors: tyrosine receptor kinases or trks (trk A, trk B, trk C) and a low-affinity receptor, "p75" (Barbacid, 1993; Chao, 1992; Glass and Yancopoulos, 1993; Meakin and Shooter, 1992). Functionally, the trks are responsible for high-affinity neurotrophin binding, leading to signal transduction, and largely mediate the biological actions of neurotrophins. In contrast, p75 (a 75-kDa glycoprotein) was originally identified as a low-affinity receptor for NGF. It was initially thought only to participate in forming the functional NGF receptor and to alter the binding affinity of trk A for NGF (Barbacid, 1993; Chao, 1992; Glass and Yancopoulos, 1993; Meakin and Shooter, 1992). Subsequent studies have shown that p75 is widely expressed by non-neuronal peripheral tissues such as Schwann cells and increases following nerve injury. In addition, p75 has been shown to act as a modifier for trk B and trk C activity as well as trk A. NGF activity is specifically associated with activation of p75 and/or trk A, with resultant effects on small sensory and autonomic nerve fibers. Large nerve fibers, in contrast, are dependent on the activity of trk C mediation of NT-3 signaling, whereas BDNF, NT-4, and NT-5 exert their effects on medium-sized fibers through the trk B high-affinity receptor.

Knockout of the p75 locus in transgenic mice was found to result in loss of small sensory ganglia, with decreased pain sensitivity and cutaneous innervation (Kuo-Fen et al., 1994). Sympathetic innervation of sympathetic chain ganglia (SCG) targeting the iris and salivary glands was not affected (Kuo-Fen et al., 1994). However, striking abnormalities in the pineal gland and the sweat glands were found. A null mutation of p75 also caused a decrease in tyrosine hydroxylase in the pineal and the footpad, which was accompanied by a reduction in pain and impaired sweating. Furthermore, the neuronal defect in p75 mutants was not due to direct effects on the target organ, but rather was due to a failure of organ development. It is not known how p75 receptor expression supports axonal growth, but it might guide developing axons or facilitate the actions of the neurotrophins at the target level. Recent studies have indicated that p75 might bind with higher affinity to proteolytic fragments from the precursor of NGF, and it is these fragments that confer pro- or antiapoptotic activity on p75 signaling (Chao and Bothwell, 2002). Alternatively, it has been suggested that p75 preferentially binds prepro-NGF in large and medium nerve fibers, whereas mature NGF signals are targeted to small fibers (Yiangou et al., 2002). An imbalance between NGF and its precursor form could result in functional deficit in the targeted neuron population.

Transgenic studies have shown that in development trk A receptor mediates neuronal survival by blocking apoptotic p75 signals (Majdan et al., 2001), emphasizing the importance of p75 interaction with trk neurotrophin receptors. Alternatively, it has been demonstrated that there is a reduction in expression of p75 in the autonomic ganglia of STZ-diabetic rats (Schmidt et al., 2000), reducing NGF signaling into the neurons. Regardless of mechanism, it is apparent that the low-affinity neurotrophin receptor is essential to the integrity of small nerve fibers involved in pain, warm thermal perception, and sweating and as such, constitute an important target for corrective therapy in diabetic neuropathy.

Contrasting with the role of the low-affinity receptor for neurotrophins in sensory and autonomic functions, it seems that the high-affinity trks may subserve motor and coordinating functions. Klein and colleagues (1994) examined the role of the trk C gene, which is expressed throughout the mammalian nervous system and encodes a series of protein kinase isoforms that serve as receptors for NT-3 (Barbacid, 1993; Emfors et al., 1992; Lamballe et al., 1991, 1993, 1994; Tessarollo et al., 1993; Tsoulfas et al., 1993). One of these isoforms, gp145$^{trkC}$/trk C K1, mediates the trophic properties of NT-3 in cultured cells. The homozygous trk C mutant mice appeared normal at birth but they grow poorly and most of them die soon after birth. Those animals that survived demonstrated motor deficits and develop athetotic movements. The trk C mutants do take nourishment and respond to painful stimuli in their whisker pads.

In contrast to mice that lack trk C, mice defective in trk B receptors (Klein et al., 1993) lose pain perception as do mice that lack trk A. Smeyne and colleagues (1994) studied the role of trks in vivo, by ablating the gene in embryonic stem cells by homologous recombination. Mice lacking trk C were found to have a severe sensory and sympathetic neuropathy and most died within 1 month of birth. They were found to have extensive neuronal loss in the trigeminal, sympathetic, and dorsal root ganglia, as well as a decrease in the cholinergic basal forebrain projections to the hippocampus and cortex. NGF induces neuron outgrowth and promotes survival of embryonic sensory and sympathetic neurons in culture, and decreases the extent of naturally occurring cell death in developing sympathetic ganglia and protects cholinergic neurons of the basal forebrain, putamen, and caudate (Bradshaw et al., 1993; Dreyfus, 1989; Levi-Montalcini, 1987). Behaviorally, the animals show reduced sensitivity to vibration and painful stimuli and have myotic pupils and ptosis. Therefore, these findings were thought to demonstrate that trk A is the primary mediator of the trophic actions of NGF in vivo, at least for certain sensory pathways (Bradshaw et al., 1993; Dreyfus, 1989; Levi-Montalcini, 1987). Hence the signaling pathway for neurotrophins via their high-affinity receptors plays a crucial role in the development of both the peripheral and central nervous systems.

How target-derived neurotrophins can send their signal back to a distal cell body through what can be a very long axon has been a question of increased study. Whether these signals are transduced locally at the cell membrane (MacInnis and Campenot, 2002) or require a signaling endosome containing both NGF and trk A (Howe et al., 2001), or likely some combination of the two, has not yet been firmly established. However, signaling from an internalized vesicle would help explain the apparent importance of retrograde transport of NGF in promoting neuronal survival.

### DIABETIC NEUROPATHY

Diabetic neuropathy comprises a number of different syndromes with a range of clinical manifestations. The main groups of neurological disturbance in diabetes include: (1) subclinical neuropathy established by abnormalities in electrodiagnostic and quanitative sensory testing; (2) diffuse clinical neuropathy with distal symmetric sensorimotor and autonomic syndromes; and (3) focal syndromes. In diabetic neuropathy, dysfunction in almost every segment of the somatic peripheral and autonomic nervous systems can be seen.

The different subcategories in diabetic neuropathy are distinguished by their pathophysiologic, therapeutic, and prognostic characteristics. The damage can influence large fibers, small fibers, or both. Small nerve fiber involvement often occurs early and may be present before objective signs or electrophysiological evidence of large fiber deficits. Small fiber loss is seen first in the lower limbs as pain and hyperalgesia, followed by loss of thermal sensitivity, and reduced light touch and pin prick sensation. A reduction of protein gene product 9.5 (PGP 9.5), SP, and CGRP in sensory neurons has been demonstrated in diabetes (Lindberger et al., 1989). PGP 9.5 is a ubiquitin hydrolase found in the cytoplasm of all nerve fibers. Studies over the last 10 years have shown a loss of epidermal nerve fibers that stain positive for PGP 9.5 in various forms of small fiber neuropathy (Holland et al., 1997). NGF is responsible for the survival and maintenance of these cutaneous fibers (Ishii et al., 1994). The reduction of NGF may be a result of down-regulation of neurofilament gene expression or of the precursor molecule of SP or CGRP, which are both down-regulated in diabetes (Ishii et al., 1994).

Patients with the most common presentation of diabetic neuropathy, distal symmetric polyneuropathy (DSPN), exhibit a "mixed" neuropathy involving both large and small nerve fibers. With DSPN, almost all patients present with a "glove and stocking" sensory loss. Large fiber dysfunction may include any combination of motor and/or sensory nerves. Large fiber loss can be seen as reduced vibration perception (often the first sign of neuropathy), or loss of position sense, weakness, muscle wasting, or depressed tendon reflexes. We have previously illustrated the relationship of different nerve fiber types, the modalities served, and the neurotrophins targeting them (Figure 1) (Vinik et al., 2003).

Diabetic neuropathy is characteristically described histopathologically with axonal degeneration, demyelination, and atrophy, in association with failed axonal regeneration, remyelination, and synaptogenesis (Calcutt et al., 1990). In recent years, there has been greater attention paid to the factors in diabetic neuropathy that may enhance nerve regeneration and protect nerves from programmed cell death. Because neurotrophins can promote the survival, maintenance, and regeneration of neurons affected in diabetes, it is likely that they play central roles in the alterations of nerve morphology and function resulting from diabetes. Supporting this notion is the amount of data showing nerve deficits as a result of removal of neurotrophins by axotomy, by depletion with experimental induction by induced autoimmunity, or failure of delivery or transport through genetic manipulation. Transgenic studies wherein the neurotrophin receptors are eliminated have confirmed and refined our appreciation of the different activities and affinities for the receptors based on neurotrophin specificities. Therefore, promotion of neurotrophin action may prove capable of preventing or reversing the changes in diabetic neuropathy. Our concepts of the role of neurotrophins and their roles in diabetic neuropathy are illustrated in Figure 2.

### NGF IN DIABETES

Data suggest that a decline in NGF synthesis in diabetes has a role in diabetic neuropathy. NGF levels in STZ-induced diabetic rats are dramatically reduced in the superior cervical ganglion, an NGF-dependent population of neurons (Steinbacher-BC and Nadelhaft 1998). Hellweg and colleagues (1991) have shown retrograde transport of NGF in the sciatic nerve to be reduced in STZ-induced diabetic rats. Decreased retrograde NGF

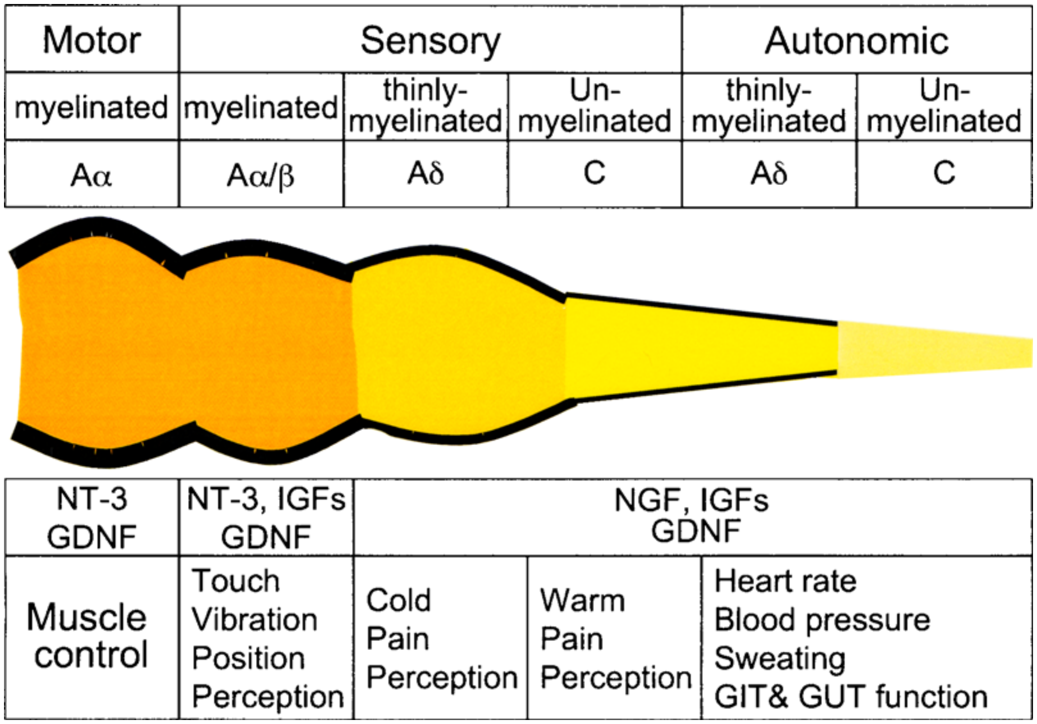

**FIGURE 1**
Schematic illustration of the peripheral nervous system, nerve fiber types, and the potential growth factors responsible for the integrity of the different fiber types (from Vinik et al., 2003).

transport in axons of the STZ-induced diabetic rat ileal mesenteric nerves has also been reported (Himes and Tessler, 1989), preceding the development of frank distal axonopathy. It seems reasonable to suspect that perturbations in NGF availability in some way contributes to the development of diabetic neuropathy. Perturbations of pain sensation are characteristic of diabetic neuropathy and the levels of SP, a nociceptive transmitter, are reduced in diabetic rats in parallel with increased tolerance to pain (Garrett et al., 1995). In addition, the amount of anterogradely transported SP was reduced in STZ-induced diabetic

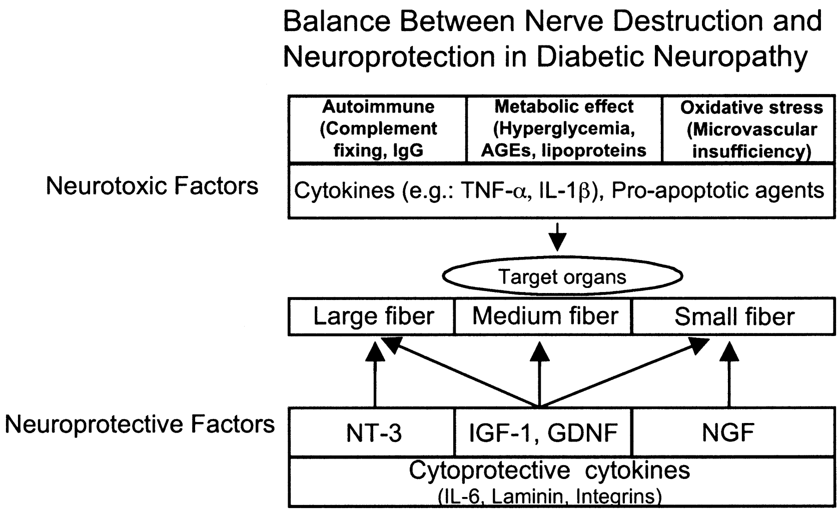

**FIGURE 2**
An illustration of the pathways to nerve damage in diabetes, the type of nerve fiber involved, and the potential for neuroprotection by growth factors, integrins, and cytokines (adapted from Vinik et al., 2003).

rats (Garrett et al., 1995; Robinson et al., 1987). Treatment with the aldose reductase inhibitor sorbinil, or its combination with gangliosides, increases the amount of SP transported in sciatic nerves of diabetic rats and is accompanied by pain hypersensitivity (Garrett et al., 1995; Robinson et al., 1987). This suggests that by increasing SP production and transport, NGF may restore disordered pain perception toward normal in diabetic neuropathy.

In normal skin, NGF is produced by basal keratinocytes, and acts via its high-affinity receptor (trk A) on nociceptor nerve fibers to increase their sensitivity, particularly in inflammation (Facer et al., 2000). In vitro studies show that keratinocytes express both NGF and its high-affinity receptor, trk A, and that NGF may increase keratinocyte proliferation and its own expression via an autocrine loop (Terenghi et al., 1997). NGF is reduced in epidermal keratinocytes in human diabetic skin, and this decrease has been related to dysfunction of cutaneous sensory fibers. These data can be interpreted to suggest that abnormal availability of target-derived NGF may be responsible in part for early small-fiber neuropathy (Anand et al., 1996). Recent data suggest that precursor forms of NGF that bind more specifically to p75 are reduced in skin from both humans and rats with diabetic neuropathy (Yiangou et al., 2002). Thus, the mechanism of target-derived NGF dysfunction at the level of the epidermis that leads to peripheral diabetic neuropathy is slowly being elucidated.

Alterations in endogenous blood concentrations of NGF may be associated with hyperglycemia and/or diabetes, and may be relevant to the development of neuropathy. Hellweg and colleagues (1991) found that endogenous NGF concentrations were low in STZ-diabetic rats and could be restored by allogenic islet transplantation. Similarly, NGF serum concentrations were diminished and tissue content was decreased in the submaxillary gland and sciatic nerve of mice made diabetic with STZ compared with matched controls (Ordoñez et al., 1994). Low levels of NGF could be due to either decreased production or transport of NGF in diabetes or both, possibly as a result of glucose-induced oxidative stress (Fernyhough et al., 1998; Hounsom et al., 2001; Stevens et al., 2000; Vincent et al., 2002). In addition, autoimmunity may play a role in the NGF deficiency in diabetes by mechanisms related to immune neutralization of available NGF. There are structural and biochemical similarities between NGF and the insulin family of peptides, and it has been suggested that antibodies to insulin may cross-react with NGF and contribute to an effective reduction in NGF available to nerves, thereby contributing to the development of neuropathy (Bennett, 1983). Because NGF-receptor signaling selectively induces tyrosine hydroxylase, and dopamine beta hydroxylase is necessary for the survival of sympathetic nerve fibers (Barde et al., 1982) and is required for the expression of SP and CGRP in adult sensory neurons, it is apparent that immune neutralization of NGF could generate a clinical syndrome not unlike that found in diabetic neuropathy (Anand et al., 1991).

### NGF IN TREATMENT OF DIABETIC NEUROPATHY

It has now been shown that NGF treatment ameliorates diabetic sensory neuropathy in animals. Apfel and colleagues (1994) administered NGF to rats at 3 $\mu$g/g or 5 $\mu$g/g three times weekly, starting 1 week after STZ administration. Reduction in pain sensation was prevented, as was the fall in CGRP in DRG in the untreated diabetic animals. NGF also prevented reduction in levels of another peptide, SP, in sensory ganglia of diabetic animals. NGF did not, however, prevent the deficit in tibial motor conduction in diabetic rats, suggesting that NGF may be a useful adjunct for the treatment of diabetic sensory and autonomic, but not motor, neuropathy (Williams et al., 1993).

Diemel and colleagues (1994) have also shown that rats with STZ-induced diabetes are depleted of both SP and CGRP peptides in the sciatic nerve, as well as having depletion of CGRP and gamma-preprotachykinin A mRNA in the fourth and fifth lumbar DRG. Treatment of rats with NGF prevented the deficits in the levels of CGRP and gamma-preprotachykinin mRNA as well as normalized the levels of CGRP and SP peptides in lumbar DRGs. The effects in the diabetic animals were much more marked than those in the nondiabetic animals. There was no response to BDNF. The demonstration that in vivo systemic administration of NGF can reverse the deficits in SP and CGRP in peripheral sensory neurons of diabetic rats lends support to the notion that deficient expression or response to NGF may be important for development of diabetic neuropathy. The authors further suggest that systemic administration of NGF may be of value in treating the sensory forms of diabetic neuropathy.

Two randomized, placebo-controlled clinical trials of recombinant human NGF (rhNGF) administered to patients with polyneuropathy were initiated. In a phase II clinical trial of rhNGF in 250 patients with diabetic polyneuropathy, improvements in signs and symptoms were seen after treatment with either 0.1 or 0.3 $\mu$g/kg rhNGF, subcutaneously, three times a week for 6 months. A second phase II trial in 270 patients with human immunodeficiency virus (HIV)-associated sensory neuropathy demonstrated significant improvements in neuropathic pain and sensitivity to pinprick, following 18 weeks of treatment with either 0.1 or 0.3 $\mu$g/kg rhNGF twice a week (Apfel et al., 1998). In addition, both studies suggested that administration of rhNGF was well tolerated with the exception of self-limited injection site hyperalgesia and other pain-related syndromes. In the phase II trial on the efficacy of rhNGF in

250 patients with diabetic neuropathy, NGF improved a composite score, which included measures of small fiber function, warm thermal threshold, cooling detection threshold, and nerve impairment scores in the lower limb (Vinik, 1999). As a result of this success, a 48-week randomized, placebo-controlled phase III study of rhNGF in a dose of 0.1 $\mu$g/kg (n = 504) or placebo (n = 515) subcutaneously 3 times/week was carried out. The primary outcome measure was change in baseline on the Neuropathy Impairment score for the lower limbs. Secondary outcome measures also included quantitative sensory test using the Case IV device, Neuropathy Symptom and Change score, and the patient benefit questionnaire. Nerve conduction studies were also performed and incidence of foot ulcers was observed. Eighty-three percent of patients completed the study, compared with 90% of the placebo group. rhNGF induced significant injection site hyperalgesia, which made true blinding difficult, and there was no significant difference in the primary outcome ($P = .25$). Furthermore rhNGF did not exhibit significant improvement in the secondary end points. However, there were significant, albeit modest, improvements in the global assessment scores ($P < .03$) and 2 of 32 comparisons in improvement in global symptoms ($P = .05$ for leg pain; $P = .003$ for 6 months' symptoms in the hands and feet). The reasons for these poor results compared with the phase II study are not entirely clear. We noted a surprising lack of progression in neuropathy in the placebo-treated group, suggesting that modern management of patients with diabetes has resulted in a change in the natural history of the condition, dictating a more robust end point and possibly longer-duration studies. If the ability of rhNGF is not robust and if all it can do is prevent the progression of neuropathy, it would not be possible to show an effect in a 1-year trial. Among the possible other reasons for the conflicting data between the phase II (Vinik, 1999) and this phase III study (Periquet et al., 1999) were possible differences in dose, concentration, and the preparation of NGF, choice of end points, measurements of neuropathy (no direct estimation of small fiber changes in the skin were made), and patient populations. Further, it is uncertain whether systemic NGF can significantly reverse microvascular defects that may contribute to neuronal loss, although it has been shown that NGF improves physiological control of microvascular perfusion (Bennett et al., 1998b). Whether this is a direct effect of NGF on microvessels or a result of improved neural function is not certain.

The end point for these studies employed a composite scale (Apfel et al., 2001) that may have masked changes specific to the nerve fibers targeted by NGF. Using sophisticated measures of cognitive function, e.g., thermal perception, requires highly skilled and trained personnel not available when advancing from a few centers in the phase II (successful) to many centers (unsuccessful) in the phase III study. With the recent developments of improved methods for identifying C fibers in skin by immunohistochemistry and quantification of nerve fiber density using the pan-neuronal marker PGP 9.5 (Herrmann et al., 1999; Langer et al., 1995; Sima and Sugimoto, 1999), and functional tests of C-fiber integrity including measures of skin blood flow (Cardillo et al., 1999; Stansberry et al., 1999), it might be possible to overcome the end point limitations inherent in the study design and rescue NGF from a premature demise as a therapeutic agent in diabetic neuropathy. Further, recent reports have demonstrated the profound effects of both microvascular dysfunction and oxidative and nitrative stress in the pathogenesis of diabetic neuropathy (Ceriello et al., 2001; Hoeldtke et al., 2002; Stuhlinger et al., 2002; Vinik et al., 2001; Zavaroni et al., 2000). It may be that neurotrophins alone cannot overcome the damage caused by these mechanisms to peripheral nerve fibers, thus requiring multimodal treatment to achieve the desired recovery.

### NGF/GROWTH FACTOR INTERACTION

NGF may also interact with other neurotrophic agents to modify neuronal survival. An example of this is the effect of IGF-II on NGF receptors. In serum-free medium, NGF receptor binding diminishes and NGF fails to have a physiological effect on the neuron as a result. Addition of IGF-II to this serum-free medium can induce NGF receptor binding and thus restore NGF function in neurons. Although IGFs and NGF can work together to maintain normal nerve integrity, there appears to be a separate role for each of these neurotrophins in neurite outgrowth. Antisera to NGF, for example, do not block the effects of IGF on tubulin synthesis and differentiation. Aside from the direct effects insulin and the IGFs have on neurite outgrowth and regeneration, it is possible that IGFs exert a major action through modulation of NGF activity.

It has been known for some time that insulin stimulates many anabolic processes in its target cells. Recently, this has been stated in a more unified concept designated as the pleiotypic response. The processes that have been found to be under pleiotypic control are uridine uptake, RNA synthesis, polysome formation, protein synthesis, protein degradation, and glucose utilization (Vinik et al., 1995b). The response to insulin closely parallels that of sensitive neurons to NGF in vitro. Sensory ganglia respond to NGF with an increase in uridine uptake, synthesis of all classes of RNA, protein synthesis, and glucose utilization. Also, NGF increases lipid synthesis, an aspect that might be related to a pleiotypic growth response (Watkins, 1995). The striking similarity between the structure of NGF and proinsulin suggests an evolution of NGF from an ancestral proinsulin. This may be an example of converging evolution with formation of a new function from a preexisting protein and may be compared with the evolution of alpha-lactalbumin from lysozyme and the development of several pancreatic serine proteases with varying specificity from a primitive precursor (Hill et al., 1968). Thus, NGF might best be considered among the growth factors as occupying a position intermediate between protein hormones and inducers. Frazier and colleagues (Frazier et al., 1972) have developed a model in which they suggest that an ancestral proinsulin comprised of B, C, and A chains finally gives rise to a fragment of 50 residues, which comprises the insulin A and B chains, undergoes contiguous reduplication, deletion, and modification, with final translation into an NGF with 118 residues, comprised of B, C, A, and B chains. The stimulating pathways or pleiotropic actions of insulin and NGF may include two distinct but related pathways involving stimulation of both serine/threonine kinases and phosphatases (Saltiel, 1990; Saltiel and Cuatrecasas, 1988). The sequence of events following the initial interaction with NGF and insulin and the receptor bear remarkable similarities. The NGF receptors bear considerable structural homology with the insulin receptor. Within the trk domain, all of the trk proteins showed 40% to 50% sequence identity with the insulin receptor and over 80% sequence similarity. The similarities between NGF and insulin in structure, cellular actions, early signaling events, and receptor structure/function have led to renewed interest in the biology of newly discovered and previously recognized neurotrophic factors. It is indeed for this reason that we are interested in the overlap between the actions and mechanisms of insulin and neurotrophins and their involvement in the pathogenesis of diabetic polyneuropathy.

### NGF AND APOPTOSIS

Recent studies indicate that apoptosis may be a mechanism whereby diabetes induces nerve damage and wherein diabetes opposes the antiapoptotic mechanisms that normally protect nerves. However, this remains to be demonstrated in human diabetes. It is of interest that NGF can rescue neuronal cells from apoptosis (Jensen et al., 1992; Rabizadeh et al., 1993). Apoptosis is regulated by many extrinsic and intrinsic cellular signals, and the threshold of apoptotic cell death is also dynamically regulated by multiple inducers and inhibitors of gene products (Steller, 1995). Several apoptosis-related oncogene products are also expressed and regulated by extracellular signals in neurons. Bcl-2, a cell-death suppressor, is found in the mitochondrial membrane, the nucleus, and the endoplasmic reticulum. A high level of expression of bcl-2 in sympathetic neurons prevented cell death induced by deprivation of NGF (Garcia et al., 1992).

Fas (APO-1, CD95) is a type I cell-surface receptor with a molecular weight of 35 to 40 kDa, dependent on the source species, belonging to the tumor necrosis factor (TNF)/NGF receptor superfamily. Fas is expressed in many cell lines, but the largest body of research shows that Fas mediates apoptosis in susceptible T-lymphocyte target cells (Weller et al., 1994). Fas-mediated apoptosis may be antibody dependent. When Fas ligand (FasL) or anti-Fas antibodies bind to the Fas receptor, the target cell undergoes apoptosis. The apoptotic signal through Fas requires the cross-linking and trimerization of Fas receptors (Peitsch and Tschopp, 1995). The trimerized Fas complex can then be activated by antibody action, resulting in the transduction of the signal for induction of apoptosis. Polymerization of Fas can be accomplished either with (FasL) or with antibodies to Fas recognizing specific epitopes (Nagata and Golstein, 1995). Consistent with this hypothesis, our studies utilizing immunofluorescence with N1E-115 neuroblastoma cells revealed a clustering of Fas on the neuroblastoma cell surface in response to type 1 diabetic serum (Pittenger et al., 1997), in contrast to its normal diffuse distribution on the cell membrane. This clustering appears to be a consequence of molecular cross-linking of Fas by a factor in type 1 diabetic serum. According to our previous study, the cytotoxic factor is likely to be an autoantibody (Pittenger et al., 1995). Our finding that type 1 diabetic serum blocks the Fas cell-surface immunofluorescence using a Fas-specific antiserum suggests competition of the immunoglobulin G (IgG) in type 1 diabetic serum and the rabbit anti-Fas antibody, indicating that Fas might be one of the membrane antigens recognized by autoantibodies in type 1 diabetic serum. Cytotoxicity of type 1 diabetic serum might be enhanced by the expression of Fas or an increase in circulating FasL. Indeed, Guillot et al. (2001) report an increase of circulating Fas, but not FasL, in patients with diabetic neuropathy, but not in diabetes patients with retinopathy or no complications. Thus, it may be that down-regulation of NGF as seen in diabetes may unbridle the action of circulating Fas in peripheral nerves, specifically linking Fas to neuronal destruction in diabetes. At this time, there is no evidence in the literature for or against the presence of Fas or FasL action on peripheral neurons in diabetes.

There is good evidence from studies in neuroblastoma cell cultures for antiapoptotic signaling through the phosphatidylinositol 3′-kinase signaling pathway (Jaboin et al., 2002), with subsequent signaling through the mitogen-activated protein (MAP) kinase/Akt cascade, resulting in neurite outgrowth (Piiper et al., 2002). If the MAP kinase/Akt signaling system is disrupted, as appears to be the case in diabetes, this could compromise NGF signaling and contribute to the development of diabetic neuropathy, in some cases even before the metabolic effects become symptomatic. We cannot exclude the possibility that other unknown antigens or death factors, such as glycolipids, the low-affinity NGF receptor (p75), the TNF-$\alpha$ receptor, or other as yet unknown regulators of apoptosis, may also be involved. The roles of these regulators in apoptotic neuronal death and whether they might contribute to the development of diabetic neuropathy remain to be elucidated. These findings support the possibility that alternate treatments for diabetic neuropathy may include antiapoptotic maneuvers.

Another possible route of antiapoptotic action for NGF is blockade of oxidative stress pathways. It has been shown that NGF can serve as an antioxidant in its own right (Lieberthal et al., 1998; Pan et al., 1997). Thus, with the reduced levels of NGF seen in diabetes, there is a concomitant increase in peroxynitrites in local tissues, leading to cleavage of caspases and activation of the apoptotic cascade (Park et al., 1998). NGF appears to exert these effects through the low-affinity p75 receptor (Vincent et al., 2002) Another aspect of NGF treatment of neurons is the up-regulation of the antiapoptotic cellular proteins bcl-2 and bcl-x (Muller et al., 1997; Park et al., 1998). Thus, there are a number of ways for neurotrophins, and NGF specifically, to promote neuronal survival in the face of diabetes.

### THERAPEUTIC POTENTIAL FOR NGF

As we understand the role of neurotrophins in the control of growth initiation, proliferation of neurons, and the apoptotic process, it becomes possible to consider the use of neurotrophic factors in the treatment of diabetic neuropathy. Aided by the availability of large quantities of recombinant neurotrophic factors, it is feasible to consider their possible place in the management of diabetic neuropathy. The choice of the optimal neurotrophic factor is dependent upon an awareness of the neuronal population involved in the disease process and an understanding of the specificity of each factor for a specific neuronal population affected by the disease process. This emphasizes the need for more specific delineation of the neuronal population involved in the disease process as well as the specific syndrome present in a particular patient. Because both sympathetic and DRG express receptors for NGF, it may prove efficacious for the treatment of both the small fiber autonomic and sensory neuropathies. The optimum approach to mixed sensory neuropathies may be the use of factors with less specificity for motor or sensory neurons. Approximately a third of primary sensory neurons do not express trk receptors, and can be identified by the binding of the lectin IB4. These neurons have been found to express Ret mRNA, the signal transduction component of the receptor for GDNF, a member of the transforming growth factor (TGF)-$\beta$ superfamily (Bennett et al., 1998a). Ret is a common receptor for GDNF and neurturin, and it interacts with either of two other receptor subunits, GFR$\alpha$-1 and GFR$\alpha$-2. Ret receptors have been identified in small-diameter primary sensory neurons, and colocalize with GFR$\alpha$-1 and GFR$\alpha$-2 subunits (Vinik et al., 1999). Initially, GDNF was thought to support survival of only dopaminergic neurons, but recent reports have shown it to be a potent neurotrophic factor for many other neuronal populations. GDNF prevents the slowing of conduction velocity that normally occurs after axotomy in a population of small-diameter DRG cells and the A fiber sprouting into lamina II of the dorsal horn (Vinik et al., 1999). GDNF has a trophic effect on motoneurons and autonomic neurons (Vinik et al., 1999) as well as Schwann cells that implicate its diverse role in promoting peripheral nerve regeneration.

Alternatively, new gene therapy methods are showing promise for delivery of neurotrophins resulting in improvements in diabetic neuropathy. Goss et al. (2002) have used cytomegalovirus (CMV) immediate-early promoter for delivery of NGF into the DRG and adipose tissue and herpes simplex latency active promoter 2 delivered into the footpad of STZ-diabetic mice, and demonstrated a block of degradation of foot sensory amplitude, a marker of the development of neuropathy. It is of interest that delivery of NT-3 with an adenovirus-based vector and of VEGF with a CMV promoter both have been shown to relieve the development of neuropathy in drug-induced diabetic rats and rabbits (Pradat et al., 2001; Schratzberger et al., 2001). These studies further support the multimodal needs for reversing peripheral nerve damage. Thus, although systemic administration of NGF, and neurotrophins in general, may prove disappointing, gene therapy might provide an effective mode of delivery for these powerful agents.

By stimulating nerve growth and regeneration as well as remodeling, administration of growth factors that target neurons may decrease the vulnerability to damage by the diabetic disease process. NGF has been considered for the treatment of Alzheimer's disease because of the loss of cholinergic neurons in this disease and the pronounced and selective trophic action of NGF upon cholinergic neurons (Hefti and Schneider, 1989). Recombinant NGF has been shown to reverse experimental cholinergic injury in animals (Goodman et al., 1953). Basic fibroblast growth factor (bFGF) and BDNF also protect cholinergic cell bodies, although less well than NGF (Anderson et al., 1988; Schwaber et al., 1991). The major brunt of diabetic neuropathy is initially borne by the long parasympathetic, cholinergic nerves and their neurons, suggesting that NGF alone, or in combination with BDNF or bFGF, may have a place in the treatment of parasympathetic neuropathy.

There is a reason to consider other prospects for growth factor therapy in diabetes. Even motoneurons may be protected from cell death. CNTF rescues motoneurons from naturally occurring cell death during chick embryo development and may retard motoneuron degeneration in the adult (Sendtner et al., 1990). Among a variety of approaches being used to enhance peripheral nerve regeneration is the manipulation of Schwann cells and the use of neurotrophic factors. Such factors include NGF and the members of the neurotrophin family, namely, BDNF, NT-3, NT-4/5, the neurokines, CNTF, leukemia inhibitory factor (LIF), and the TGF-$\beta$ family and their distant relative, GDNF (Hayakawa et al., 1994).

Early results of treatment of toxic neuropathies with growth factors are encouraging. The small fiber sensory neuropathy induced by Taxol can be prevented by the administration of NGF (Konings et al., 1994). The large fiber neuropathy induced by the antitumor agent cisplatin with prominent proprioceptive deficits can be prevented in rodents treated with NGF (Hiraiwa et al., 1997), which has also been shown to prevent or delay the development of sensory neuropathy in STZ-induced diabetes.

### SUMMARY

Although the mechanism of action is as yet unknown, the current knowledge of growth factors, and NGF specifically, and their relationship to diabetic neuropathy suggests a pathophysiological role for reduced levels of NGF available for retrograde transport to neuronal cell bodies. In addition, there is likely a compromise of the p75 and trk A receptors responsible for mediating NGF signaling. It is now conceivable that neuronal function may be compromised, and atrophy of nerves, and possibly even cell death, may be a consequence of a reduction of overall NGF activity in diabetic neuropathy. Whether the growth factor deficiency is due to decreased synthesis, an inability of the factor to bind to its receptor, disturbances in retrograde axonal transport, or intraneuronal processing also remains to be established. Further studies aimed at understanding the disturbances in expression of NGF-related genes and proteins, as well as their receptor binding and subsequent transport from sites of synthesis to sites of action should shed considerable light on the relationship between NGF activity and diabetic neuropathy. Other contributors to the pathogenesis of diabetic neuropathy, such as microvascular disease and nitrative stress, will also have to be addressed in any further attempts to use neurotrophic factor therapy. It is unlikely that neurotrophins will succeed as monotherapy, but their use in conjunction with agents that combat the myriad of dysfunctions in diabetes is likely to yield better results. Discovery of these relationships will hopefully lead to more efficacious therapies involving neurotrophins in general and NGF specifically.

### REFERENCES

Anand, P., Rudge, P., Mathias, C. J., Springall, D. R., Chatel, M. A., Naher-Noe, M., Sharief, M., Misra, V. P., Polak, J. M., Bloom, S. R., and Thomas, P. K. (1991) New autonomic and sensory neuropathy with loss of adrenergic sympathetic function and sensory neuropeptides. *Lancet,* **337,** 1253–1254.

Anand, P., Terenghi, G., Warner, G., Kopelman, P., Williams Chestnut, R. E., and Sinicropi, D. V. (1996) The role of endogenous nerve growth factor in human diabetic neuropathy. *Nat. Med.,* **2,** 703–707.

Anderson, K. J., Dam, D. Lee, S., and Cotman, C. W. (1988) Basic fibroblast growth factor prevents death of lesioned cholinergic neurons in vivo. *Nature,* **332,** 360–361.

Apfel, S. C., Arezzo, J. C., Brownlee, M., Federoff, H., and Kessler, J. A. (1994) Nerve growth factor administration protects against experimental diabetic sensory neuropathy. *Brain Res.,* **634,** 7–12.

Apfel, S. C., Asbury, A., Bril, V., Burns, T., Campbell, J., Chalk, C., Dyck. P., Dyck, P. J., Feldman, E., Fields, H., Grant, I., Griffin, J., Klein, C., Lindblom, U., Litchy, W., Low, P., Melanson, M., Mendell, J., Merren, M., O'Brien, P., Rendell, M., Rizza, R., Service, F., Thomas, P. K., Walk, D., Wang, A., Wessel, K., Windebank, A., Ziegler, D., and Zochodne, D. (2001) Positive neuropathic sensory symptoms as endpoints in diabetic neuropathy trials. *J. Neurol. Sci.,* **189,** 3–5.

Apfel, S. C., Kessler, J. A., Adornato, B. T., Litchy, W. J., Sanders, C., Rask, C. A., and the NGF Study Group. (1998) Recombinant human nerve growth factor in the treatment of diabetic polyneuropathy. *Neurology,* **51,** 695–702.

Barbacid, M. (1993) Structural and functional properties of TRK family of neurotrophin receptors. *Ann. N.Y. Acad. Sci.,* **766,** 442–458.

Barde, E., Edgar, D., and Thoenen, H. (1982). Purification of a new neurotrophic factor from mammalian brain. *EMBO J.,* **1,** 549–553.

Benbow, S. J., Pryce, D. W., Noblett, K., MacFarlane, I. A., Friedmann, P. S., and Williams, G. (1995) Flow motion in peripheral diabetic neuropathy. *Clin. Sci.,* **88,** 191–196.

Bennett, D. L., Michael, G. J., Ramchandran, N., Munson, J. B., Averill, S., yan, Q., McMahon, S. B., and Priestley, J. V. (1998a) A distinct subgroup of small DRG cells express GDNF receptor components and GDNF is protective for these neurons after nerve injury. *J. Neurosci.,* **18,** 3059–3072.

Bennett, G. S., Garrett, N. E., Diemel, L.T., Brain, S. D., and Tomlinson, D. R. (1998b) Neurogenic cutaneous vasodilatation and plasma extravasation in diabetic rats: Effect of insulin and nerve growth factor. *Br. J. Pharmacol.,* **124,** 1573–1579.

Bennett, T. (1983). Physiological investigation of diabetic autonomic failure. In: *Autonomic Failure. A Textbook of Clinical Disorders of the Autonomic Nervous System,* Edited by Bannister, R., pp. 406–436. Oxford, UK, Oxford University Press.

Bradshaw, R. A., Blundell R. L., Lapato, R., McDonald, N. Q., and Murray-Rust, J. (1993) Nerve growth factor revisited. *Trends Biochem. Sci.,* **18,** 48–52.

Calcutt, N. A., Tomlinson, D. R., Willars, G., and Keen, P. (1990) Axonal transport of substance P-like immunoreactivity in ganglioside-treated diabetic rats. *J. Neurol. Sci.,* **96,** 283–291.

Caputo, G. M., Cavanagh, P. R., Ulbrecht, J. S., Gibbons, G. W., and Karchmer, A. W. (1994) Assessment and management of foot disease in patients with diabetes. *N. Engl. J. Med.,* **331,** 854–860.

Cardillo, C., Nambi, S., Kilcoyne. C., Choucair, W., Katz, A., Quon, M., and Panza, J. (1999) Insulin stimulates both endothelin and nitric oxide activity in the human forearm. *Circulation,* **100,** 820–825.

Ceriello, A., Mercuri, F., Quagliaro, L., Assaloni, R., Motz, E., Tonutti, L., and Taboga, C. (2001) Detection of nitrotyrosine in the diabetic plasma: Evidence of oxidative stress. *Diabetologia,* **44,** 834–838.

Chao, M. V. (1992) Neurotrophin receptors: A window into neuronal differentiation. *Neuron,* **9,** 583–593.

Chao, M. V., and Bothwell, M. (2002) Neurotrophins: To cleave or not to cleave. *Neuron*, **33,** 9–12.

DCCT Research Group. (1993) The effect of intensive treatment of diabetes on the development and progression of long-term complications in insulin dependent diabetes mellitus. *N. Engl. J. Med.,* **329,** 977–986.

Diemel, L. T., Brewster, W. J., Fernyhough, P., and Tomlinson, D. R. (1994) Expression of neuropeptides in experimental diabetes: Effects of treatment with nerve growth factor or brain-derived neurotrophic factor. *Mol. Brain Res.,* **21,** 171–175.

Dreyfus, C. F. (1989) Effects of nerve growth factor on cholinergic brain neurons. *Trends Pharmacol. Sci.,* **10,** 145–149.

Emfors, P., Merlio, J. P., and Persson, H. (1992) Cells expressing messenger RNA for neurotrophins and their receptors during embryonic rat development. *Eur. J. Neurosci.,* **4,** 1140–1158.

Facer, P., Mann, D., Mathur, R., Pandya. S., Ladiwala, U., Singhal, B., Hongo, J., Sinicropi, D. V., Terenghi, G., and Anand, P. (2000) Do nerve growth factor-related mechanisms contribute to loss of cutaneous nociception in leprosy? *Pain,* **85,** 231–238.

Fernyhough, P., Brewster, W. J., Fernandes, K., Diemel, L. T., and Tomlinson, D. R. (1998) Stimulation of nerve growth-factor and substance P expression in the iris-trigeminal axis of diabetic rats–involvement of oxidative stress and effects of aldose reductase inhibition. *Brain Res.,* **802,** 247–253.

Frazier, W. A., Hogue-Angeletti, R., and Bradshaw, R. A. (1972) Nerve growth factor and insulin, structural similarities indicate an evolutionary relationship reflected by physiological action. *Science,* **176,** 482–488.

Garcia, I., Martinou, I., Tsujimoto, Y., and Martinou, J. C. (1992) Prevention of programmed cell death of sympathetic neurons by the bcl-2 proto-oncogene. *Science,* **258,** 302–304.

Garrett, N. E., Kidd, B. L., Cruwys, S. C., and Tomlinson, D. R. (1995) Streptozotocin-induced diabetes decreases substance P levels in experimental arthritis in the rat knee. *Neurosci. Lett.,* **187,** 201–204.

Glass, D., and Yancopoulos, G. D. (1993) Neurotrophins and their receptors. *Trends Cell Biol.,* **3,** 262–268.

Goodman, J. I., Baumoel, S., and Frankel, L. (1953) *The Diabetic Neuropathies*. Springfield, IL, Charles C. Thomas.

Goss, J. R., Goins, W. F., Lacomis, D., Mata, M., Glorioso, J. C., and Fink, D. J. (2002) Herpes simplex-mediated gene transfer of nerve growth factor protects against peripheral neuropathy in streptozotocin-induced diabetes in the mouse. *Diabetes,* **51,** 2227–2232.

Greene, D. A., Sima, A. A., Stevens, M. J., Feldman, E.L., and Lattimer, S. A. (1992) Complications: Neuropathy, pathogenetic considerations. *Diabetes Care,* **15,** 1902–1925.

Guillot, R., Bringuier, A. F., Porokhov, B., Guillausseau, P. J., and Feldmann, G. (2001) Increased levels of soluble Fas in serum from diabetic patients with neuropathy. *Diabetes Metab.,* **27,** 315–321.

Hallbook, F., Ibanez, C. F., and Persson, H. (1991) Evolutionary studies of the nerve growth factor family reveal a novel member abundantly expressed in Xenopus ovary. *Neuron,* **6,** 845–858.

Hayakawa, K., Sobue, G., Itoh, T., and Mitsuma, T. (1994) Nerve growth factor prevents neurotoxic effects of cisplatin, vincristine and taxol, on adult rat sympathetic ganglion explants in vitro. *Life Sci.,* **55,** 519–525.

Hefti, F., and Schneider, L. S. (1989) Rationale for the planned clinical trials with nerve growth factor in Alzheimer's disease. *Psychiatr. Dev.,* **7,** 297–315.

Hellweg, R., Wohrle, M., and Hartung, H. D. (1991) Diabetes mellitus associated decrease in nerve growth factor levels is reversed by allogenetic pancreatic islet transplantation. *Neurosci. Lett.,* **125,** 1–4.

Herrmann, D. N., Griffin, J. W., and Hauer, P. (1999) Intraepidermal nerve fiber density, sural nerve morphometry and electrodiagnosis in peripheral neuropathies. *Neurology,* **53,** 1634–1640.

Hill, R. L., Brew, K., Vanaman, T. C., Trayer, I. P., and Mattock, P. (1968) Function, and evolution of alpha-lactalbumin. *Brookhaven Symp. Biol.,* **21,** 139–154.

Himes, B.T., and Tessler, A. (1989) Death of some dorsal root ganglion neurons and plasticity of others following sciatic nerve section in adult and neonatal rats. *J. Comp. Neurol.,* **284,** 215–230.

Hiraiwa, M., Martin, B., Kishimoto, Y., Conner, G., Tsuji, S., and O'Brien, J. (1997) Lysosomal proteolysis of prosaposin, the precusor of saposins (Sphingolipid Activator Proteins): Its mechanism and inhibition by ganglioside. *Arch. Biochem. Biophys.,* **341,** 17–24.

Hoeldtke, R. D., Bryner, K. D., McNeil, D. R., Hobbs, G. R., Riggs, J. E., Warehime, S. S., Christie, I., Ganser, G., and Van, Dyke, K. (2002) Nitrosative stress, uric acid, and peripheral nerve function in early type 1 diabetes. *Diabetes,* **51,** 2817–2825.

Hohn, A., Leibrock, J., Bailey K., and Barde, Y. A. (1990) Identification and characterization of a novel member of the nerve growth factor/brain-derived neurotrophic factor family. *Nature,* **344,** 339–341.

Holland, N. R., Stocks, A., Hauer, P., Cornblath, D. R., Griffin, J. W., and McArthur, J. C. (1997) Intraepidermal nerve fiber density in patients with painful sensory neuropathy. *Neurology,* **48,** 708–711.

Hounsom, L., Corder, R., Patel, J., and Tomlinson, D. R. (2001) Oxidative stress participates in the breakdown of neuronal phenotype in experimental diabetic neuropathy. *Diabetologia,* **44,** 424–428.

Howe, C. L., Valletta, J. S., Rusnak, A. S., and Mobley, W. C. (2001) NGF signaling from clathrin-coated vesicles: Evidence that signaling endosomes serve as a platform for the Ras-MAPK pathway. *Neuron,* **32,** 801–814.

Ishii, D., Guertin, D. M., and Whalen, L. R. (1994) Reduced insulin-like growth factor-I mRNA content in liver, adrenal glands and spinal cord of diabetic rats. *Diabetologia,* **37,** 1073–1081.

Jaboin, J., Kim, C. J., Kaplan, D. R., and Thiele, C. J. (2002) Brain-derived neurotrophic factor activation of TrkB protects neuroblastoma cells from chemotherapy-induced apoptosis via phosphatidylinositol 3′-kinase pathway. *Cancer Res.,* **62,** 6756–6763.

Jensen, L. M., Zhang, Y., and Shooter, E. M. (1992) Steady-state polypeptide modulations associated with nerve growth factor (NGF)-induced terminal differentiation and NGF deprivation-induced apoptosis in human neuroblastoma cells. *J. Biol. Chem.,* **267,** 19325–19333.

Jones, K., and Reichardt, L. (1990) Molecular cloning of a human gene that is a member of the nerve growth factor family. *Proc. Natl. Acad. Sci. U. S. A.,* **87,** 8060–8064.

Klein, R., Silos-Santiago, I., Smeyne, R., Lira, S., Brambilla, R., Bryant, S., Zhang, L., Snider, W. D., and Barbacid, M. (1994) Disruption of the neurotrophin-3 receptor gene trkC eliminates Ia muscle afferents and results in abnormal movements. *Nature,* **368,** 249–251.

Klein, R., Smeyne, R. J., Wurst, W., Long, L. K., Auerbach, B. A., Joyner, A. L., and Barbacid, M. (1993) Targeted disruption of the trkB neurotrophin receptor gene results in nervous system lesions and neonatal death. *Cell,* **75,** 113–122.

Konings, P. N., Makkink, W. K., van Delft, A. M., and Ruigt, G. S. (1994) Reversal by NGF of cytostatic drug-induced reduction of neurite outgrowth in rat dorsal root ganglia in vitro. *Brain Res.,* **640,** 195–204.

Kuo-Fen, L., Bachman, K., Landis, S., and Jaenisch, R. (1994) Dependence on p75 for innervation of some sympathetic targets. *Science,* **263,** 1447–1449.

Lamballe, F., Klein, R., and Barbacid, M. (1991) trkC, a new member of the trk family of tyrosine protein kinases, is a receptor for neurotrophin-3. *Cell,* **66,** 967–979.

Lamballe, F., Smeyne, R., and Barbacid, M. (1994) Developmental expression of trkC, the neurotrophin-3 receptor, in the mammalin nervous system. *J. Neurosci.,* **14,** 14–28.

Lamballe, F., Tapley, P., and Barbacid, M. (1993) trkC encodes multiple neurotrophin-3 receptors with distinct biological properties and substrate specificities. *EMBO J.,* **12,** 3083–3094.

Langer, A., Freeman, M. R., Josse, R. G., and Armstrong. P. W. (1995) Metaiodobenzylguanidine imaging in diabetes mellitus: Assessment of cardiac sympathetic denervation and its relation to autonomic dysfunction and silent myocardial ischemia. *J. Am. Col. Cardiol.,* **25,** 610–618.

Levi-Montalcini, R. (1987) The nerve growth factor: Thirty-five years later. *EMBO J.,* **6,** 1145–1154.

Levi-Montalcini, R., and Booker, B. (1960) Destruction of the sympathetic ganglia in mammals by an antiserum to the nerve-growth promoting factor. *Proc. Natl. Acad. Sci. U. S. A.,* **42,** 384–390.

Levi-Montalcini, R., and Hamburger, V. (1951) Selective growth stimulating effect of mouse sarcoma on the sensory and sympathetic nervous system of the chick embryo. *J. Exp. Zool.,* **116,** 321–363.

Lieberthal, W., Triaca, V., Koh, J. S., Pagano, P. J., and Levine, J. S. (1998) Role of superoxide in apoptosis induced by growth factor withdrawal. *Am. J. Physiol.,* **275(5 Pt 2),** F691–F702.

Lindberger, M., Schroder, H. D., and Schultzberg, M. (1989) Nerve fibre studies in skin biopsies in peripheral neuropathies. I. Immunohistochemical analysis of neuropeptides in diabetes mellitus. *J. Neurol. Sci.,* **93,** 289–296.

Lindsay, R. M., and Harmar, A. J. (1989) Nerve growth factor regulates expression of neuropeptide genes in adult sensory neurons. *Nature,* **337,** 362–364.

MacInnis, B. L., and Campenot, R. B. (2002) Retrograde support of neuronal survival without retrograde transport of nerve growth factor. *Science,* **295,** 1536–1539.

Maisonpierre, P. C., Belluscio, L., Squinto, S., Ip, N. Y., Furth, M. E., Lindsay, R. M., and Yancopoulos, G. D. (1990) Neurotrophin-3: A neurotrophic factor related to NGF and BDNF. *Science,* **247,** 1446–1451.

Majdan, M., Walsh, G. S., Aloyz, R., and Miller, F. D (2001) TrkA mediates developmental sympathetic neuron survival in vivo by silencing an ongoing p75NTR-mediated death signal. *J. Cell Biol.,* **155,** 1275–1285.

Meakin, S., and Shooter, E. (1992) The nerve growth factor family of receptors. *Trends Neurosci.,* **15,** 323–331.

Melville, S., Sherburn, T. E., and Coggeshall, R. E. (1989) Preservation of sensory cells by placing stumps of transected nerve in an impermeable tube. *Exp. Neurol.,* **105,** 311–315.

Merhi, M., Dusting, G. J., and Khalil, Z. (1998) CGRP and nitric oxide of neuronal origin and their involvement in neurogenic vasodilatation in rat skin microvasculature. *Br. J. Pharmacol.,* **123,** 863–868.

Muller, Y., Tangre, K., and Clos, J. (1997) Autocrine regulation of apoptosis and bcl-2 expression by nerve growth factor in early differentiating cerebellar granule neurons involves low affinity neurotrophin receptor. *Neurochem. Int.,* **31,** 177–191.

Nagata, S., and Golstein, P. (1995) The Fas death factor. *Science,* **267,** 1449–1456.

Nakhooda, A. F., Like, A. A., Chappel, C. I., Murray, F. T., and Marliss, E. B, (1977) The spontaneously diabetic Wistar rat. Metabolic and morphologic studies. *Diabetes*, **26,** 100–112.

Nakhooda, A. F., Like, A. A., Chappel, C. I., Wei, C. N., and Marliss, E. B (1978) The spontaneously diabetic Wistar rat (the "BB" rat). Studies prior to and during development of the overt syndrome. *Diabetologia,* **14,** 199–207.

Ordoñez, G., Fernandez, A., Perez, R., and Sotelo, J. (1994) Low contents of nerve growth factor in serum and submaxillary gland of diabetic mice. A possible etiological element of diabetic neuropathy. *J. Neurol. Sci.,* **121,** 163–166.

Pan, Z., Sampath, D., Jackson, G., Werrbach-Perez K., and Perez-Polo, R. (1997) Nerve growth factor and oxidative stress in the nervous system. *Adv. Exp. Med. Biol.,* **429,** 173–193.

Park, D. S., Morris. E. J., Stefanis, L., Troy, C. M., Shelanski, M. L., Geller, H. M., and Greene, L. A. (1998) Multiple pathways of neuronal death induced by DNA-damaging agents, NGF deprivation, and oxidative stress. *J. Neurosci.,* **18,** 830–840.

Peitsch, M. C., and Tschopp, J. (1995) Comparative molecular modelling of the Fas-ligand and other members of the TNF family. *Mol. Immunol.,* **32,** 761–772.

Periquet, M. I., Novak, V., Collins, M. P., Nagaraja, H. N., Erdem, S., Nash, S. M., Freimer, M. L., Sahenk, Z., Kissel, J. T., and Mendell, J. R. (1999) Painful sensory neuropathy: Prospective evaluation using skin biopsy. *Neurology,* **53,** 1641–1647.

Pierson, C. R., Zhang, W., Murakawa, Y., and Sima, A. A. (2003) Insulin deficiency rather than hyperglycemia accounts for impaired neurotrophic responses and nerve fiber regeneration in type 1 diabetic neuropathy. *J. Neuropathol. Exp. Neurol.,* **62,** 260–271.

Piiper, A., Dikic, I., Lutz, M. P., Leser, J., Kronenberger, B., Elez, R., Cramer, H., Muller-Esterl, W., and Zeuzem, S. (2002) Cyclic AMP induces transactivation of the receptors for epidermal growth factor and nerve growth factor, thereby modulating activation of MAP kinase, Akt, and neurite outgrowth in PC12 cells. *J. Biol. Chem.,* **277,** 43623–43630.

Pittenger, G. L., Liu, D., and Vinik, A. I. (1995) The neuronal toxic factor in serum of type 1 diabetic patients is a complement-fixing autoantibody. *Diabetic Med.,* **12,** 380–386.

Pittenger, G. L., Liu, D., and Vinik, A. I. (1997) The apoptotic death of neuroblastoma cells caused by serum from patients with insulin-dependent diabetes and neuropathy may be Fas-mediated. *J. Neuroimmunol.,* **76,** 153–160.

Pradat, P. F., Kennel, P., Naimi-Sadaoui, S., Finiels, F., Orsini, C., Revah, F., Delaere, P., and Mallet, J. (2001) Continuous delivery of neurotrophin 3 by gene therapy has a neuroprotective effect in experimental models of diabetic and acrylamide neuropathies. *Hum. Gene Ther.,* **12,** 2237–2249.

Rabizadeh, S., Oh, J., Zhong, L. T., Yang, J., Bitler, C. M., Butcher, L. L., and Bredesen, D. E. (1993) Induction of apoptosis by the low-affinity NGF receptor. *Science,* **261,** 345–348.

Rich, K. M., Luszczynski, J. R., Osborne, P. A., and Johnson, E. M., Jr. (1987) Nerve growth factor protects adult sensory neurons from cell death and atrophy caused by nerve injury. *J. Neurocytol.,* **16,** 261–268.

Robinson, J. P., Willars, G. B., Tomlinson, D. R., and Keen, P. (1987) Axonal transport and tissue contents of substance P in rats with long-term streptozotocin-diabetes. Effects of the aldose reductase inhibitor 'statil.' *Brain Res.,* **426,** 339–348.

Rosenthal, A., Goeddel, D. V., Nguyen, T., Lewis, M., Shih, A., Laramee, G. R., Nikolics, K., and Winslow, J. W. (1990) Primary structure and biological activity of a novel human neurotrophic factor. *Neuron,* **4,** 767–773.

Saltiel, A. R. (1990) Signal transduction in insulin. *J. Nutr. Biochem.,* **1,** 180–188.

Saltiel, A. R., and Cuatrecasas, P. (1988) In search of a second messenger for insulin. *Am. J. Physiol.,* **255,** C1–C11.

Sango, K., Verdes, J. M., Hikawa, N., Horle, H., Tanaka, S. I., Inoue, S., Sotelo, J. R., and Takenaka, T. (1994) Nerve growth factor (NGF) restores depletions of calcitonin gene-related peptide and substance P in sensory neurons from diabetic mice in vitro. *J. Neurol. Sci.,* **126,** 1–5.

Schmidt, R. E., Dorsey, D. A., Roth, K. A., Parvin, C. A., Hounsom, L., and Tomlinson, D. R. (2000) Effect of streptozotocin–induced diabetes on NGF, P75(NTR) and TrkA content of prevertebral and paravertebral rat sympathetic ganglia. *Brain Res.,* **867,** 149–156.

Schmidt, Y., Unger, J. W., Bartke, I., and Reiter, R. (1995) Effect of nerve growth factor on peptide neurons in dorsal root ganglia after taxol or cisplatin treatment and in diabetic (db/db) mice. *Exp. Neurol.,* **132,** 16–23.

Schratzberger, P., Walter, D. H., Rittig, K., Bahlmann, F. H., Pola, R., Curry, C., Silver, M., Krainin, J. G., Weinberg, D. H., and Ropper, A. H. (2001) Reversal of experimental diabetic neuropathy by VEGF gene transfer. *J. Clin. Invest.,* **107,** 1083–1092.

Schwaber, J. S., Due, B. R., Rogers, W. T., Junard, E. O., and Hefti, F. (1991) Use of a digital brain atlas to compare the distribution of NGF- and bFGF-protected cholinergic neurons. *J. Comp. Neurol.,* **309,** 27–39.

Schwartz, J. P., Pearson, J., and Johnson, E. M (1982) Effect of exposure to anti-NGF on sensory neurons of adult rats and guinea pigs. *Brain Res.,* **244,** 378–381.

Sendtner, M., Kreutzberg, G. W., and Thoenen, H. (1990) Ciliary neurotrophic factor prevents the degeneration of motor neurons after axotomy. *Nature,* **345,** 440–441.

Sima, A. A., Zhang, W., Sugimoto, K., Henry, D., Li, Z., Wahren, J., and Grunberger, G. (2001) C-peptide prevents and improves chronic Type I diabetic polyneuropathy in the BB/Wor rat. *Diabetologia,* **44,** 889–897.

Sima, A. A. F., and Sugimoto, K. (1999) Experimental diabetic neuropathy: An update. *Diabetologia,* **42,** 773–788.

Smeyne, R. J., Klein, R., Schnapp, A., Long, L. K., Bryant, S., Lewin, A., Lira, S. A., and Barbacid, M. (1994) Severe sensory and sympathetic neuropathies in mice carrying a disrupted Trk/NGF receptor gene. *Nature,* **368,** 246–249.

Stansberry, K. B., Peppard, H. R., Babyak, L. M., Popp, G., McNitt, P. M., and Vinik, A. I. (1999) Primary nociceptive afferents mediate the blood flow dysfunction in non-glabrous (hairy) skin of type 2 diabetes. *Diabetes Care,* **22,** 1549–1554.

Stansberry, K. B., Shapiro, S. A., Hill, M. A., McNitt, P. M., Meyer, M. D., and Vinik, A. I. (1996) Impaired peripheral vasomotion in diabetes. *Diabetes Care,* **19,** 715–721.

Steinbacher-BC, J., and Nadelhaft, I. (1998) Increased levels of nerve growth factor in the urinary bladder and hypertrophy of dorsal root ganglion neurons in the diabetic rat. *Brain Res.,* **782,** 255–260.

Steller, H. (1995) Mechanism and genes of cellular suicide. *Science,* **267,** 1445–1449.

Stevens, M. J., Obrosova, I., Cao, X., Van Huysen, C., and Greene, D. A. (2000) Effects of DL-alpha-lipoic acid on peripheral nerve conduction, blood flow, energy metabolism, and oxidative stress in experimental diabetic neuropathy. *Diabetes,* **49,** 1006–1015.

Stuhlinger, M. C., Abbasi, F., Chu, J. W., Lamendola, C., McLaughlin, T. L., Cooke, J. P., Reaven, G. M., and Tsao, P. S. (2002) Relationship between insulin resistance and an endogenous nitric oxide synthase inhibitor. *JAMA,* **11,** 1420–1426.

Terenghi, G., Mann, D., Kopelman, P. G., and Anand, P. (1997) trkA and trkC expression is increased in human diabetic skin. *Neurosci. Lett.,* **228,** 33–36.

Tessarollo, L., Tsoulfas, P., Martin-Zanca, D., Gilbert, D. J., Jenkins, N. A., Copeland N.G., and Parada, L. F. (1993) trkC, a receptor for neurotrophin-3, is widely expressed in the developing nervous system and in non-neuronal tissues. *Development,* **118,** 463–475.

Tsoulfas, R., Soppet, D., Escandon, E., Tessarollo, L., Mendoza-Ramirez, J. L., Rosenthal, A., Nikolics, K., and Parada, L. F. (1993) The rat trkC locus encodes multiple neurogenic receptors that exhibit differential response to neurotrophin-3 in PC 12 cells. *Neuron,* **10,** 975–990.

Vincent, A., Brownlee, M., and Russell, J. (2002) Oxidative stress and programmed cell death in diabetic neuropathy. *Ann. N.Y. Acad. Sci.,* **959,** 368–383.

Vinik, A. I. (1999) Treatment of diabetic polyneuropathy (DPN) with recombinant human nerve growth factor (rhNGF). *Diabetes,* **48(Suppl 1),** A54–A55.

Vinik, A. I., Erbas, T., Park, T., Stansberry, K. B., Scanelli, J. A., and Pittenger, G. L. (2001) Dermal neurovascular dysfunction in type 2 diabetes. *Diabetes Care,* **24,** 1486–1475.

Vinik, A. I., Mitchell, B. D., Leichter, S. B., Wagner, A. L., O'Brian, J. T., and Georges, L. P. (1995a) Epidemiology of the complications of diabetes. In: *Diabetes: Clinical Science in Practice*, Edited by Leslie, R. D. G., and Robbins, D. C., pp. 221–287. Cambridge, UK, Cambridge University Press.

Vinik, A. I., Newlon, P. G., Lauterio, T. J., Liuzzi, F. J., Depto A. J., Pittenger, G. L., and Richardson, D. W. (1995b) Nerve survival and regeneration in diabetes. *Diabetes Metab. Rev.,* **3,** 139–157.

Vinik, A. I., Pittenger, G. L., Milicevic, Z., and Knezevic-Cuca, J. (1999) Autoimmune mechanisms in the pathogenesis of diabetic neuropathy. In: *Molecular Mechanisms for Endocrine and Organ Specific Autoimmunity*, Edited by Eisenbarth, R. G., pp. 217–251. Georgetown, Landes.

Vinik, A. I., Pittenger, G., Stansberry, K., Park, T. S., and Erbas, T. (2003) Neurotrophic factors. In: *Textbook of Diabetic Neuropathy*, Edited by Gries, F. A., Low, P. A., Cameron, N. E., and Ziegler, D., pp. 129–169. Stuttgart, Germany, Georg Thieme Verlag.

Watkins, A. D. (1995) Perceptions, emotions and immunity: An integrated homoeostatic network. *Q. J. Med.,* **88,** 283–294.

Weller, M., Frei, K., Groscurth, P., Krammer, P. H., Yonekawa, Y., and Fontana, A. (1994) Anti-Fas/APO-1 antibody-mediated apoptosis of cultured human glioma cells. Induction and modulation of sensitivity by cytokines. *J. Clin. Invest.,* **94,** 954–964.

Williams, L. M., Dell, C., and Stramm, L. E. (1993) 306159 inhibits agonist induced alterations in retinal capillary endothelial cell proteoglycan metabolism. *Diabetes,* **42(Suppl. 1),** 158A.

Yiangou, Y., Facer, P., Sinicropi, D. V., Boucher, T. J., Bennett, D. L., McMahon, S. B., and Anand, P. (2002) Molecular forms of NGF in human and rat neuropathic tissues: Decreased NGF precursor-like immunoreactivity in human diabetic skin. *J. Peripher. Nerv. Syst.,* **7,** 190–197.

Zavaroni, I., Platti P. M. L., Gasparini, P., Barilli, L., Massironi, P., Ardigo, D., Valsecchi, G., Delsignore, R., and Reaven, G. (2000) Plasma nitric oxide concentrations are elevated in insulin-resistant healthy projects. *Metabolism,* **49,** 959–961.

